# The moderating role of perceived injustice in the relationship between being envied and social undermining in public sector organizations

**DOI:** 10.3389/fpsyg.2025.1582051

**Published:** 2025-06-10

**Authors:** Mohammad Yarivand, Hend Faye Al-shahrani, Mohammad Ahmed Hammad, Meraj Malakouti

**Affiliations:** ^1^Public Administration, Huazhong University of Science and Technology, Wuhan, China; ^2^Social Service Department, College of Humanities and Social Sciences, Princess Noura Bint Abdul Rahman University, Riyadh, Saudi Arabia; ^3^Department of Education and Psychology, Najran University, Najran, Saudi Arabia; ^4^Department of Education and Psychology, Assiut University, Assiut, Egypt

**Keywords:** social undermining, envy, perceived injustice, disruptive behavior, workplace mistreatment

## Abstract

**Purpose:**

Social undermining in the workplace—subtle behaviors that hinder success and damage professional relationships—has been widely studied. However, the role of envy in driving social undermining, particularly from the perspective of the person being envied, remains underexplored. This study examines how being envied influences social undermining behaviors and tests whether perceived injustice moderates this relationship within Tehran’s public sector.

**Methods:**

A cross-sectional design with purposive sampling was employed. A web-based questionnaire was completed by 342 full-time Iranian civil servants. Data were analyzed using path analysis and bootstrapping procedures to test direct and moderated relationships.

**Results:**

Being the target of envy was positively associated with social undermining, and this relationship was significantly strengthened under conditions of high perceived injustice. Furthermore, higher levels of social undermining correlated with increased psychological distress and lower life satisfaction.

**Conclusion:**

The findings demonstrate the destructive impact of envy and perceived injustice on employees in Iranian governmental organizations. Organizations should implement fairness-enhancing policies and interventions to mitigate envy-driven undermining and safeguard employee well-being.

## 1 Introduction

Social undermining, characterized by subtle actions intended to damage a colleague’s work or reputation, negatively impacts morale, productivity, and employee well-being, frequently leading to anxiety, depression, and diminished life satisfaction (Mulaphong, 2022; Howard et al., 2020; Tan and Xia, 2021; Costa et al., 2024; Haider et al., 2025). While envy is recognized as a significant precursor to interpersonal deviance (Smith and Kim, 2007), existing research has largely concentrated on the motivations behind undermining behaviors, rather than examining the experiences and responses of those who are envied. This oversight is especially significant in public-sector contexts, where established hierarchies and restricted advancement opportunities heighten concerns regarding fairness.

Field studies indicate that when colleagues view an individual as a threat to their status, envy may incite interpersonal deviance and tactics aimed at undermining that individual. Targets of envy experience increased occurrences of malicious gossip, rumor dissemination, and passive-aggressive conduct (Lee and Duffy, 2019; Haider et al., 2025). While social undermining has been thoroughly investigated since the concept was popularized by Duffy et al. (2020), the majority of research focuses on the motivations of perpetrators and organizational outcomes, resulting in a relative lack of examination of the victim’s experience. In public organizations characterized by strict hierarchies and limited resources, employees frequently view reward and recognition processes as inequitable, leading to feelings of resentment (Duffy et al., 2006). Perceptions of injustice can amplify the destructive effects of envy, leading to increased undermining behaviors directed at individuals perceived as more fortunate (Ye et al., 2021; Li et al., 2021). Previous studies indicate that injustice not only predisposes individuals to engage in undermining behaviors but also increases sensitivity to being targeted, consequently exacerbating mental health outcomes (Haider et al., 2025). Civil servants’ mental health is a critical concern; stressors such as high public scrutiny and job insecurity heighten vulnerability to anxiety and depression (Howard et al., 2020; Tan and Xia, 2021). Few studies examine the relationship between interpersonal envy, social undermining, and mental health outcomes in public-sector contexts.

This study connects these constructs to address a theoretical gap regarding the interplay of emotion and fairness perceptions, as well as a practical gap in identifying risks to employee well-being. We propose and test a model in which being envied predicts social undermining, with perceived injustice serving as a moderator, and examine how these dynamics jointly affect civil servants’ mental health. Specifically, we ask:

1. How does being envied influence social undermining among Tehran’s public-sector employees?
2. To what extent does perceived injustice moderate this relationship?
3. What are the downstream effects on anxiety, depression, and life satisfaction?

The public sector in Tehran, with hierarchical organizations and scarce resources, is a unique context through which these processes can be investigated. Tehran’s civil servants work under significant levels of pressure with scarce resources at their disposal, with the added dimension of having to compete for recognition and advancement. These conditions can escalate perceptions of injustice and envy, feeding interpersonal tension as well as social undermining. By investigating these processes, this research hopes to contribute toward the improved understanding of how Tehran’s public sector workplaces contribute toward the employees’ mental health. Also, the research is significant in the improvement of the mental health of Tehran’s civil servants, with the view of affecting the organizations’ practices in similar workplaces all across the globe.

## 2 Literature review and hypothesis

### 2.1 Definition of workplace mistreatment

Workplace mistreatment encompasses a range of abusive behaviors directed at employees, which adversely affect their health and performance (Cullen et al., 2014). Behaviors encompass verbal abuse, physical assault, bullying, prejudice, and emotional harm (Burns, 2022). Social undermining is a significant form of mistreatment, characterized as behaviors aimed at obstructing a coworker’s capacity to develop and sustain positive relationships, achieve work success, and maintain a favorable reputation over time (Mulaphong, 2022). Social undermining has significant repercussions, including increased psychological distress, diminished job satisfaction, and decreased performance (Meier and Cho, 2019; Haider et al., 2025). Victims of social undermining also show a greater vulnerability to mental health disorders, particularly anxiety and depression (Costa et al., 2024).

Recent empirical research indicates that envy is a significant motivator of social undermining, which can stem from various sources. Shoukat et al. (2025) discovered that frontline hotel employees exhibiting high levels of workplace envy engaged in significantly greater service-sabotage behaviors, a type of social undermining, compared to their peers with lower levels of envy. In a separate field study, researchers discovered that malicious envy stemming from upward social comparisons increased employees’ propensity to disseminate rumors and employ other undermining strategies against envied colleagues (Li and Wang, 2023). Recent findings highlight that envy, defined as resentment towards individuals perceived as more fortunate or accomplished, serves as a significant driver of social undermining in various organizational contexts.

Given the complexity of workplace mistreatment, it is therefore critical to consider how individual emotions like envy interact with broader organizational factors, such as perceived injustice, to exacerbate social undermining—particularly in public-sector contexts where hierarchical rigidity and resource scarcity heighten fairness concerns.

### 2.2 Envy, perceived injustice and social undermining among civil servants

Envy arises when people engage in unfavorable social comparisons, being envious about people whom they see as more fortunate or more accomplished (Haider et al., 2025). In the work environment of the public sector organizations, envy is highly relevant because the work environment is highly competitive with limited advancement chances. Negative behavior like social undermining can be evoked through envy since employees’ sabotage or undermine the accomplishment of their fellow employees with the aim of eliminating perceived disparities (Reh et al., 2018; Song and Zhao, 2022). Prior work in organizational behavior has predominantly examined the drivers of social undermining as a form of counterproductive behavior, focusing on why individuals enact undermining (e.g., Meier and Cho, 2019; Haider et al., 2025). This study focuses on the recipient’s perspective, examining how experiences of envy and perceptions of injustice influence the experience of social undermining. This shift in focus—from perpetrator to target—fills a significant gap in the literature and corresponds with calls for increased attention to the effects of envy on its subjects (Song and Zhao, 2022; Khairy et al., 2025).

Social Identity Threat Theory is a useful conceptual framework explaining how envy is expressed through work behavior. According to the theory, when employees see their social identity as under attack, they can counter with unfavorable work behavior like social undermining with the aim of defending their self-esteem (Johnson et al., 2024). Research has demonstrated threats can create a hostile work environment, with employees who envy their fellow employees more likely to engage in actions like exclusion, sabotage, and gossip (Khairy et al., 2025). Perceived injustice, or feeling mistreated at work, can escalate the aversive emotional responses created by envy (Adamovic, 2023). As per the Organizational Justice Theory, mistreatment is considered justifying revenge because people seek to restore fairness (Adamovic, 2023). In the public sector, ambiguity in the bureaucracy (e.g., unclear procedures for advancement) amplifies perceptions of injustice, provoking envy-driven anger toward rival colleagues (Boon and Brown, 2020). For example, Li et al. (2021) found employees who felt mistreated would be more inclined toward justifying sabotaging rival colleagues as morally acceptable. This process is highly applicable in the case of the civil service, as limited professional advancement opportunities create heightened competition as well as heightened attention toward fairness (Sullivan et al., 2012; Iverson et al., 2018).

In order to preserve theoretical parsimony and ensure that any observed moderation effect can be attributed unambiguously to justice perceptions, this study restricts its core model to perceived injustice as the sole boundary condition for the link between being envied and social undermining. Previous studies have looked at a great variety of other mediators and control factors, showing their effect on workplace abuse (e.g., Song and Zhao, 2022; Haider et al., 2025). The current study, on the other hand, emphasizes only perceived injustice so as not to dilute the particular value of fairness judgements in a non-Western, public-sector setting. Therefore, the following assumptions according the conceptual model:

*Hypothesis 1 (H1)*: Being Envied is positively related to being undermined among civil servants.

*Hypothesis 2 (H2)*: Feelings of injustice (Perceived injustice) moderate the positive relation of being envied and being undermined among civil servants.

### 2.3 Mental health implications of social undermining at work

Social undermining at work harms the employees’ work performance as well as their well-being but also bears significant mental health ramifications. Individuals seek to safeguard valuable resources such as self-esteem as well as well-being in a bid to cope with the work pressures as well as achieve their work objectives as per the Conservation of Resources (COR) model (Hobfoll et al., 2018). Employees who experience social undermining lose these vital resources, thereby developing higher levels of psychological distress as well as lower levels of life satisfaction (D'Ambrosio et al., 2018; Song and Zhao, 2022).

**Figure 1:**
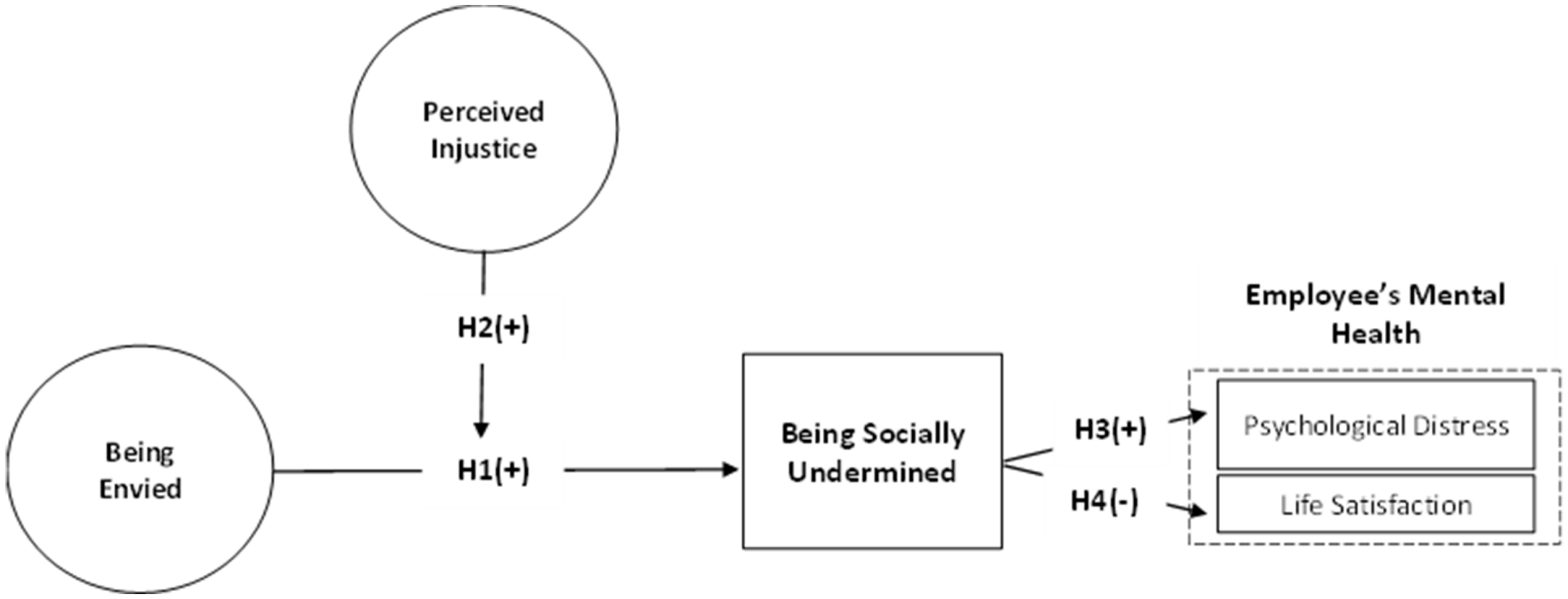
Conceptual model.

Social undermining is more destructive when applied in the context of public sector organizations since job uncertainty and excessive competition for resources create the conditions under which bad behavior can manifest. Employees who have been socially undermined can be more prone to job uncertainty, anxiety, as well as feeling hopeless, all contributing to their low well-being (Mulaphong, 2022; Bohle et al., 2022). Consequently, the psychological effect of social undermining can be the catalyst for impaired job performance, low job motivation, as well as heightened levels of tension, thus developing a vicious cycle affecting the individual as well as the organization (Figure 1). Thus, the following hypotheses are advanced:

*Hypothesis 3 (H3)*: Being undermined at work will be associated with higher levels of psychological distress among victims.

*Hypothesis 4 (H4)*: Being undermined at work will be associated with lower life satisfaction among victims.

## 3 Materials and methods

### 3.1 Procedure and participants

This study used a cross-sectional design with purposive sampling to choose individuals based on particular characteristics. Purposive sampling was chosen to ensure that participants from public organizations, specifically civil servants in non-managerial roles, were represented in the sample. Particularly in Iran’s public-sector setting, where hierarchical systems and restricted development prospects made focused sampling vital, this method proved well-suited to capture the subtle dynamics of envy and perceived injustice.

This study employed a web-based survey approach, targeting 700 full-time civil servant employees without managerial responsibilities in a Tehran-based governmental telecommunication organization. Given its key influence on Iran’s economy and the people’s dependence on its services, the selection of this organization was intentional. From 2021 to 2022, online self-report surveys translated from English to Persian using a back-and-forth approach to guarantee linguistic and cultural equivalency gathered data. The survey was administered online, and participants were recruited via email. The email addresses of the participants were obtained through the cooperation of the human resources department of the targeted governmental organization, which provided a list of employees who met the study’s inclusion criteria. Participants were sent an email invitation explaining the study’s purpose, voluntary participation, and confidentiality of their responses. A unique link to the survey was included in the email, and no identifying information was collected to maintain participant anonymity. The survey yielded a commendable 49% response rate, resulting in 342 completed surveys. Because data were collected at a single point in time, causal direction among envy, injustice perceptions, and social undermining cannot be definitively established. Future research employing longitudinal or experimental designs is recommended to verify temporal and causal relationships. Participants consisted mainly of males (52%) between the ages of 35 and 44, with over 48% possessing bachelor’s degrees and having tenure exceeding 4 years. Table 1 presents the description of the sample.

**Table 1:** Demographics of the respondents.

Items	Frequency	Percentage
Age		
18–24	39	11.4
25–34	119	34.8
35–44	140	40.9
45–55	37	10.8
+55	7	2.1
Gender		
Female	161	47.1
Male	181	52.9
Marital		
Single	131	38.3
Married	211	61.7
Children		
No	174	50.9
Yes	168	49.1
Education		
Diploma	14	4.1
Associate’s degree	89	26
Bachelor’s Degree	166	48.5
Master’s Degree	66	19.3
Ph. D.	7	2.1
Sleep quality		
Very Poor	54	15.8
Poor	63	18.4
Fair	137	40.1
Good	88	25.7
Sports activity		
No	187	54.7
Yes	155	45.3
Tenure		
Below 3 years	81	23.7
Over 4 years	261	76.3
Overtime work		
No	143	41.8
Yes	199	58.2
Working hour		
<=48	173	50.6
49–60	131	38.3
+61	38	11.1

Independent-samples *t*-tests were employed to compare scale scores between early (*n* = 168) and late (*n* = 174) respondents, while chi-square tests were utilized for demographic comparisons. No significant differences were found for Being Envied [*t*(340) = −0.75, *p* = 0.45], Perceived Injustice [*t*(340) = 0.38, *p* = 0.70], or Social Undermining [*t*(340) = 1.02, *p* = 0.31]. Chi-square tests indicated no significant association between response timing and Age bracket [χ^2^(4) = 2.84, *p* = 0.58], Gender [χ^2^(1) = 0.18, *p* = 0.67], Education level [χ^2^(3) = 2.45, *p* = 0.29], or Tenure category [χ^2^(1) = 1.98, *p* = 0.57], thereby supporting representativeness.

To test robustness across subgroups, we conducted multi-group analyses by Gender (male vs. female) and Age (younger: categories 1–2 [18–34] vs. older: categories 3–5 [35+]). Following configural, metric, and scalar invariance procedures, the measurement model demonstrated adequate invariance across both sets of groups. Structural invariance tests indicated no significant differences in the key path coefficients (Being Envied → Perceived Injustice; Perceived Injustice → Social Undermining) across gender [Δχ^2^ (2) = 3.10, *p* = 0.21] or age groups [Δχ^2^ (2) = 2.75, *p* = 0.25], suggesting that the hypothesized relationships remain consistent across these demographics.

### 3.2 Measures

#### 3.2.1 Being envied

The three-item scale by Lee et al. (2018) measures employees’ perceptions of being envied by coworkers. A sample item is “Because of my success at work; I am sometimes envied by my coworkers” (*a* = 0.94). Each item included a 7-point Likert scale (anchors: 1 = very inaccurate, 7 = very accurate) (*α* = 0.653).

#### 3.2.2 Perceived injustice

Perceived injustice was assessed using a 20-item questionnaire by Colquitt et al. (2015). Participants rated items on a 7-point Likert scale, and the questionnaire had previously been translated into Persian with a Cronbach’s alpha of 0.94 (Kakemam et al., 2021). An example item is “To what extent are outcomes insufficient, given the work you have completed?.” (*α* = 0.908).

#### 3.2.3 Social undermining experience

Social undermining experience was assessed with four items: “In the past 2 months, have you experienced any of the following acts of workplace aggression/harassment that we directed at you? (a) spreading rumors or negative comments about you to undermine your status; (b) undeserved criticisms; (c) unreasonable assignments or deadlines; and (d) sabotaging performance (Mulaphong, 2022).” These items resembled Duffy et al. (2012) Social Undermining scale. Responses were coded 1 (Never), 2 (Once), and 3 (More than once). (*α* = 0.763).

#### 3.2.4 Psychological distress

The GHQ-12, created by Goldberg and Williams (1988) as a brief measure of psychological distress, is widely used in non-clinical settings (Tomás et al., 2015). It has been translated into various languages, including Farsi (Namjoo et al., 2017). Higher GHQ-12 scores indicate worse mental well-being. Sample questions from the GHQ-12 include: “Have you been able to concentrate on your tasks in the past few weeks?” and “Have you felt capable of making decisions recently?” (α = 0.771).

#### 3.2.5 Life satisfaction

Diener et al. (1985) developed a five-item, seven-point Likert scale to assess life satisfaction, with higher scores indicating more pleasure. Bayani et al. (2007) investigated the reliability and validity of a Persian adaption of this measure. Their evaluation yielded a robust Cronbach’s alpha value of 0.83 for internal consistency and a respectable test–retest reliability of 0.69 (α = 0.895).

#### 3.2.6 Control variables

Based on Gharibi et al. (2016), this paper used a control variable framework comprising socio-demographic factors (age, gender, marital status, children, educational level), health-related variables (exercise activity, sleep quality), and work-related factors (job tenure, overtime work, work hours). Demographic controls were included in Step 1 of the hierarchical regressions. Gender (0 = female; 1 = male), marital status (0 = single; 1 = married), parental status (0 = no children; 1 = children), sports activity (0 = no; 1 = yes), and overtime (0 = no; 1 = yes) were included as dichotomous covariates. Age, education level, tenure, sleep quality, and working hours were classified as ordinal variables based on the categories presented in Table 2 (e.g., age: 1 = 18–24, 2 = 25–34, …, 5 = > 55). This coding produces the reported means (e.g., M_age = 2.57, SD_age = 0.90) and guarantees that each control is accurately modelled.

**Table 2:** Descriptive statistics and correlations among all variables.

Variable	M ± SD	1	2	3	4	5	6	7	8	9	10	11	12	13	14	15
Age	2.57 ± 0.9	1														
Gender	1.53 ± 0.5	0.03	1													
Marital	1.62 ± 0.49	0.08	−0.03	1												
Children	1.49 ± 0.5	−0.01	0.1	−0.2*	1											
Education	3.89 ± 0.85	−0.04	−0.07	0.04	0.03	1										
Sleep	2.76 ± 1.01	−0.12*	−0.02	−0.06	0.05	−0.21**	1									
Sport	1.45 ± 0.5	−0.01	0.05	−0.02	−0.01	0.13*	−0.09	1								
Tenure	1.76 ± 0.43	0.04	−0.06	0.09	−0.11*	0	−0.05	0.07	1							
Overtime	1.58 ± 0.49	−0.03	0	−0.01	−0.01	−0.21**	0.09	−0.01	0.07	1						
Workh	1.61 ± 0.68	−0.02	−0.06	0.04	−0.04	0.04	0.02	−0.1	−0.06	−0.16**	1					
BE	3.21 ± 1.2	−0.02	−0.06	0	−0.03	0.12*	−0.08	0.1	0.04	−0.09	0.02	1				
SU	1.44 ± 0.48	−0.03	0.01	0.04	−0.08	0.14**	−0.04	0.08	−0.01	−0.09	0.01	0.59**	1			
PI	3.12 ± 0.91	−0.02	−0.05	0.08	−0.09	0.18**	−0.1	0.13*	0.05	−0.12*	0.02	0.69**	0.66**	1		
PD	1.88 ± 0.35	−0.06	0.03	0.01	−0.05	0.15**	−0.06	0.13*	−0.01	−0.07	0.04	0.62**	0.58**	0.75**	1	
SWL	5.21 ± 1.43	0.04	−0.06	0.02	−0.04	−0.31**	0.25**	−0.14**	0.01	0.1	0.05	0.15**	0.21**	0.21**	0.17**	1

*N* = 342. *p < 0.05, **p < 0.01. 1 = Age; 2 = Gender; 3 = Marital status; 4 = Children; 5 = Education; 6 = Sleep; 7 = Sport; 8 = Tenure; 9 = Overtime; 10 = Work hours; 11 = BE (Being envied); 12 = SU (Social undermining); 13 = PI (Perceived injustice); 14 = PD (Psychological distress); 15 = SWL (Satisfaction with life).

### 3.3 Data analysis

This study conducted a preliminary statistical analysis of the data using SPSS. Subsequently, the validity of the proposed hypotheses was assessed through the application of Partial Least Squares Structural Equation Modeling (PLS-SEM). PLS-SEM was chosen as the appropriate method due to the explanatory nature of this research and the observation that the data did not adhere to a normal distribution, as evidenced by the results of the Shapiro–Wilk test (refer to Table 3). The selection of PLS-SEM was underpinned by its capability to examine a theoretical framework from a predictive perspective, as articulated by Hair et al. (2019). Additionally, PLS-SEM was preferred over AMOS as it optimizes the explained variance in the dependent variable(s) and provides greater statistical power across all sample sizes, aligning with the recommendations of Hair et al. (2017a).

**Table 3:** Results for confirmatory factor analysis.

Models	CMIN/DF	RMR (SRMR)	GFI	TLI	CFI	RMSEA
Five-factor model (BE; SU; PI; PD; SWL)	2.75	0.075	0.91	0.90	0.92	0.065
Four-factor model (BE+SU; PI; PD; SWL)	3.05	0.079	0.90	0.89	0.90	0.075
Three-factor model (BE+SU + PI; PD; SWL)	3.20	0.082	0.89	0.87	0.88	0.080
Two-factor model (BE+SU + PI+PD; SWL)	3.30	0.085	0.89	0.86	0.87	0.082
Single-factor model	4.20	0.110	0.88	0.75	0.77	0.095

### 3.4 Common method bias

This research applied single-source self-reported measures, which carries the risk of common method bias (CMB) (Podsakoff and Organ, 1986). Measures were put in place to mitigate this, involving the collection of the measures at more than one point in time as well as keeping the participants anonymous as well as their responses. Harman’s single-factor test, a validated method (Podsakoff et al., 2003), was utilized to evaluate the impact of common method bias (CMB) on validity. Principal component extraction without rotation (Podsakoff et al., 2003) indicated a single factor accounting for only 25.752% of the variance, significantly below the 50% threshold. This ensures the validity as well as research findings were free from the impact of CMB, affirming the validity of the study.

To provide a more rigorous assessment, we then conducted a series of confirmatory factor analyses (CFAs) comparing our hypothesized five-factor measurement model against increasingly constrained models, culminating in a single-factor solution (Table 3). The five-factor model (BE; SU; PI; PD; SWL) demonstrated good fit (χ^2^/df = 2.75; SRMR = 0.075; RMSEA = 0.065; CFI = 0.92; TLI = 0.90; GFI = 0.91), indicating that our constructs are empirically distinct. In contrast, the single-factor model showed poor fit across all indices (χ^2^/df = 4.20; SRMR = 0.110; RMSEA = 0.095; CFI = 0.77; TLI = 0.75; GFI = 0.88), further ruling out a pervasive common-method factor.

### 3.5 Reliability and validity tests

This study followed the guidelines set by Hair et al. (2017a) and conducted a thorough evaluation of the measurement model’s quality, including reliability, internal consistency, and validity. Internal consistency was assessed using two measures: Composite Reliability and Cronbach’s alpha. Notably, all obtained values exceeded the 0.6 threshold recommended by Nunnally and Bernstein (1994), confirming the model’s strong internal consistency (see Table 4).

**Table 4:** Measurement model results.

Construct	CR	AVE	*R* ^2^	*Q* ^2^	α	Shapiro–Wilk statistic
BE	0.813	0.593			0.653	0.949**
SU	0.849	0.585	0.496	0.274	0.763	0.812**
PI	0.92	0.577			0.908	0.987**
PD	0.822	0.596	0.307	0.084	0.771	0.983**
SWL	0.921	0.701	0.038	0.024	0.895	0.876**
$(\mathrm{GFI})=\sqrt{\bar{\mathrm{AVE}}\times \bar{R^{2}}}$		0.413				

Convergent validity assessment involved examining two key indicators: the Average Variance Extracted (AVE) and the outer loadings of constructs. It is important to note that all values for these indicators surpassed the established minimum threshold of 0.4, following the criteria delineated by Henseler et al. (2009). Factor loadings were generally above 0.7, except for a few cases, notably PI8, PI12, PI13, PI15, and PI19, which fell slightly below 0.6. Furthermore, discriminant validity was verified through Heterotrait-Monotrait (HTMT) analysis, revealing correlations among study variables well below the recommended threshold value of 0.85 (Table 5), thus affirming the distinctiveness of our constructs, in line with Henseler et al. (2015).

**Table 5:** Discriminant validity–heterotrait-monotrait ratio (HTMT).

Construct	BE	PD	PI	SU	SWL	PI x BE
BE						
PD	0.807					
PI	0.817	0.811				
SU	0.868	0.658	0.756			
SWL	0.254	0.38	0.391	0.224		
PI x BE	0.348	0.191	0.163	0.323	0.344	

BE = Being envied; SU = Social undermining; PI = Perceived injustice; PD = Psychological distress; SWL = Satisfaction with life.

## 4 Results

### 4.1 Descriptive results

The outcomes of the statistical analysis and the relationships among the research variables are shown in Table 2. Among the fundamental research variables, notable relationships were found. BE showed notable positive links with both SU and PI, implying a close connection between workplace envy and views of social undermining and justice (*r* = 0.59, *p* < 0.001; *r* = 0.69, *p* < 0.001). Additionally, SU demonstrated a correlation with PD and SWL (*r* = 0.58, *p* < 0.001; *r* = 0.21, *p* < 0.001), indicating possible negative impacts on psychological well-being and life satisfaction. Education level exhibited a positive relationship with envy, perceived injustice, and social undermining; however, its small yet significant negative correlation with life satisfaction (*r* = −0.31, *p* < 0.001) merits further examination. A possible explanation is that civil servants with higher education possess heightened expectations regarding career advancement and job rewards. When these expectations are not fulfilled—often seen in inflexible public-sector hierarchies—they report decreased satisfaction (Pita and Torregrosa, 2023). The overqualification or mismatch effect illustrates that education can concurrently exacerbate envy, due to the visibility of success, while also diminishing well-being when career progression halts. Moreover, Sleep Quality showed a positive link with SWL (*r* = 0.25, *p* < 0.001), hence supporting the known connection between sleep quality and general well-being. On the other hand, overtime work showed a negative association with Perceived Justice (PI; *r* = −0.12, *p* < 0.05), suggesting that more overtime might lead to worse impressions of equality and justice in the office.

### 4.2 Hypothesis tests

After validating the outer model, a rigorous path analysis was conducted following the guidelines by Hair et al. (2017b). A non-parametric bootstrapping approach with 5,000 resamples was used in this investigation to determine route coefficients and t-values meticulously. The model’s performance was comprehensively understood by examining R2, Q2, and f2, offering insights beyond the traditional reliance on *p*-values alone (Hair et al., 2017b). Table 6 shows the detailed findings of the structural model.

**Table 6:** Structural model results.

Relationships	Path coefficient	*t*	*p*	*f* ^2^	VIF
BE - > SU	0.297	4.099	0.000	0.295	1.841
SU - > PD	0.554	13.596	0.000	0.443	1.000
SU - > SWL	−0.196	2.882	0.004	0.201	1.000
PI × BE - > SU	0.141	3.067	0.002	0.241	1.096

BE = Being envied; SU = Social undermining; PI = Perceived injustice; PD = Psychological distress; SWL = Satisfaction with life.

**Figure 2:**
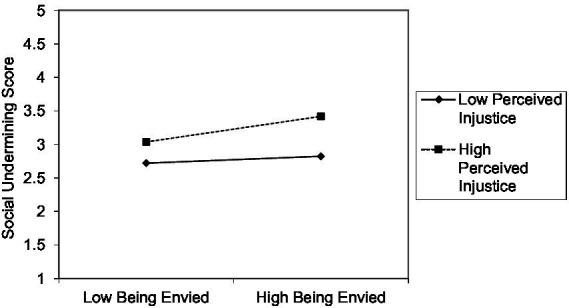
Perceived injustice moderates the effect of being envied on social undermining.

**Figure 3:**
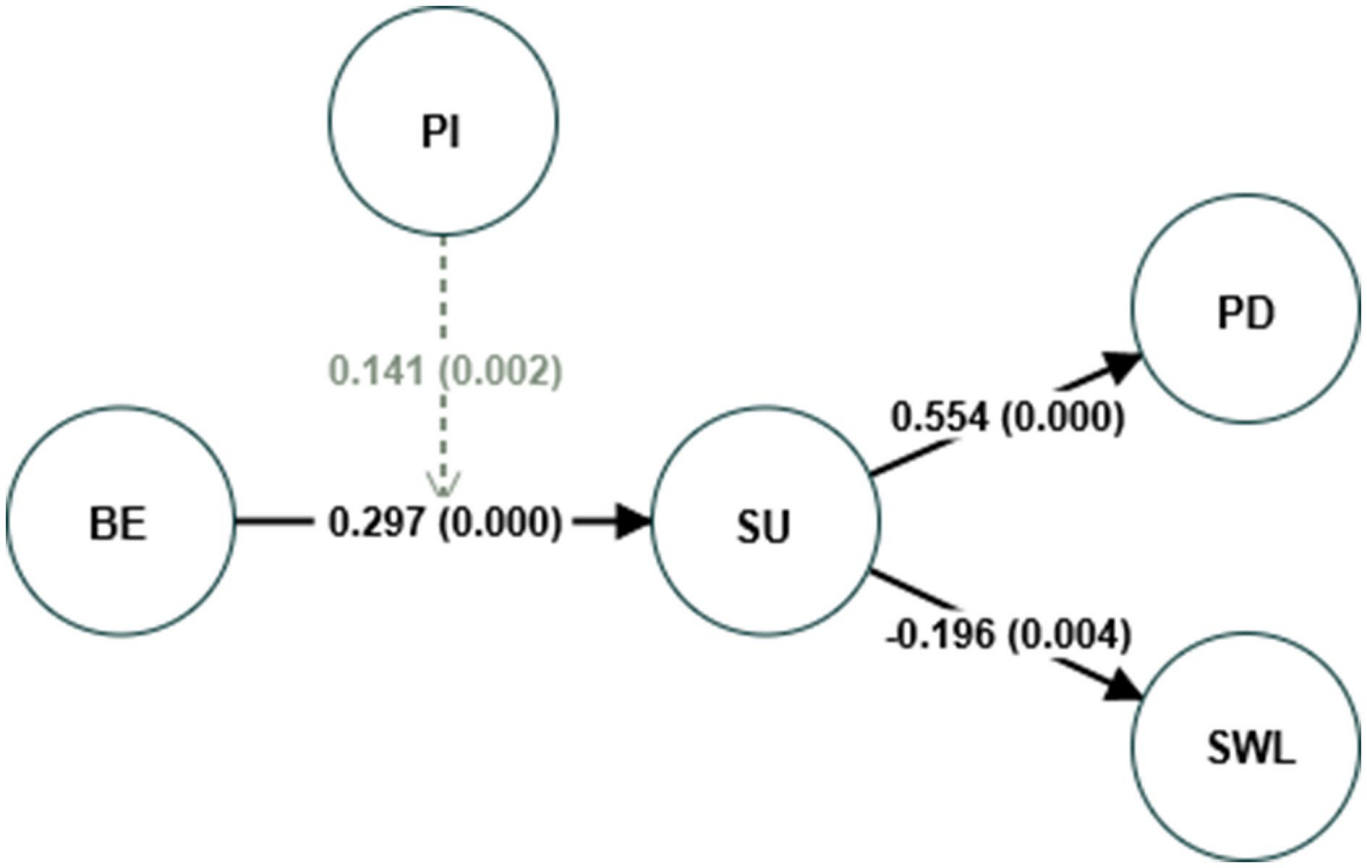
Model test results.

The findings of this investigation reveal a significant positive relationship between BE and SU (*β* = 0.297, *t* = 4.099, *p* = 0.000, *f*2 = 0.295), providing robust support for H1. Additionally, SU had a significant positive impact on PD (*β* = 0.554, *t* = 13.596, *p* = 0.000, *f*2 = 0.443), thereby corroborating the hypothesis posited in H3. Furthermore, SU exhibited a significant adverse effect on SWL (*β* = −0.196, *t* = 2.882, *p* = 0.004, *f*2 = 0.201), aligning with the expectations outlined in H4. A two-stage approach, as recommended by Henseler and Fassot (2010) for its superior statistical power, was adopted to explore the moderating effect of PI. The normalized data show that the PI_BE on SU interaction terms (*β* = −0.141, *t* = 3.067, *p* = 0.002, *f*2 = 0.241) are statistically significant at the *p* 0.001 level, with modest effect sizes. This finding lends strong support to H2. Following Dawson (2014) guidance, two-way interaction effects were also plotted in this research (Figure 2), vividly illustrating the moderating role of PI in the relationship between BE and SU. The graph highlights a more pronounced impact of BE on SU in high PI conditions (Figure 3).

Tenenhaus et al. (2005) recommended using a goodness-of-fit index (GFI) to assess overall model fit. The computed GFI value of 0.413 is above the set threshold of 0.36 for a high impact size, supporting the appropriateness of the model fit as proposed by Cohen (1988). In addition, the blindfolding method with an omission distance of 5 was used to evaluate predictive significance. This investigation produced a Q2 value more significant than zero, suggesting the model’s ability to generate correct predictions, as described by Hair et al. (2017b).

## 5 Discussion

This study has highlighted the importance of covert workplace mistreatment, focusing on interpersonal envy and undermining behaviors. Notably, this research focuses on governmental organizations in Iran, a developing nation. It is worth mentioning that such covert workplace activities have yet to get much attention from the public administration area. The empirical work of the study supported all the hypotheses.

The positive link between experiencing envy and encountering undermining behaviors confirms that envy-driven colleague actions often precede social undermining. This finding aligns with Duffy et al.'s (2020) research on the contagious nature of negative workplace behaviors. The literature on workplace mistreatment highlights the detrimental effects of such behavior on interpersonal relationships and group dynamics within organizations. Envy induces actions that undermine trust and collaboration among colleagues. Furthermore, when individuals perceive injustice in the workplace, the effects of feeling undermined are exacerbated, as indicated by a notable interaction. This emphasizes the need to consider contextual factors in understanding mistreatment dynamics (Hershcovis et al., 2020). In unjust workplaces, envy-driven actions can lead to more pronounced social undermining. The literature presents inconsistent results concerning the relationship between envy and undermining behavior. A meta-analysis of workplace envy indicates that the outcomes of envy differ significantly depending on context and measurement methods (Howard et al., 2020). Several studies reveal non-significant direct effects on counterproductive behavior when job resources and personality controls are accounted for (Li et al., 2021). Mao et al. (2022) conducted a daily diary study involving Chinese employees and discovered that envy predominantly forecasted minor counterproductive behaviors, including diminished effort and minor acts of incivility, rather than explicit social undermining. Tai et al. (2022) found that the influence of envy on interpersonal deviance was diminished in teams characterized by strong collective identification, indicating additional factors beyond mere envy effects. Cross-cultural research indicates that collectivist norms may suppress overt expressions of envy, resulting in more subtle forms of negative behavior (Li et al., 2021). In numerous organizational contexts characterized by leadership that endorses ethical practices or teams that possess ample resources, the direct relationship between envy and undermining appears to weaken. By contrast, in the non-Western public-sector environment examined here—marked by rigid hierarchies and a strong focus on procedural fairness—perceived injustice sharply heightens envy’s tendency to translate into social undermining.

The current research discovered that the Iranian public sector is now exhibiting undermining practices, which are less noticeable. Being mistreated at work might cause victims unfavorable effects, which is consistent with earlier studies done in the private sector. Reduced self-efficacy and job satisfaction and increased health complaints (Costa et al., 2024), heightened stress (Booth et al., 2019), Increased employee hostility (Hongbo et al., 2019), Increased employee silence (Jung and Yoon, 2019), increased employee counterproductive behaviors, withdrawal, and turnover intentions (Duffy et al., 2006) are all associated with undermining behaviors. Notably, a positive correlation is established between undermining experiences and Psychological Distress, highlighting the psychological toll inflicted by social undermining (Costa et al., 2024). This highlights the need for governmental organizations to address and mitigate these behaviors to preserve employees’ well-being. On the other hand, undermining experiences also negatively impact life satisfaction and lower general quality of life. Moreover, our findings contribute to existing research by showing that workplace undermining is linked to a decline in victims’ mental health. The deterioration of perceived agency support and workplace collegiality (Einarsen et al., 1994) fosters perceptions of injustice and unfair interpersonal treatment (Sarnecki et al., 2024). The identified dynamics diminish trust and collaboration, which in turn lowers the likelihood of whistleblowing intentions (Ugaddan and Park, 2019), heightens disengagement from work responsibilities (e.g., absenteeism, lateness), and ultimately results in voluntary resignation (Schneider et al., 1997). This study highlights the significant effects of workplace interpersonal undermining, which can be as detrimental as explicit abusive conduct. Organizations that emphasize a respectful and inclusive work culture are more likely to improve employee well-being and overall workplace productivity.

### 5.1 Theoretical and managerial implications

This study makes a valuable contribution to the literature by confirming the presence of covert mistreatment behaviors, social undermining, in the Iranian public sector. The research highlights that insidious mistreatment can be as perilous as overt mistreatment, resonating with current literature (Duffy et al., 2012; Jung and Yoon, 2019; Booth et al., 2019; Hongbo et al., 2019). This contradicts and expands traditional wisdom on workplace mistreatment, calling for the need to recognize and address subtle and pervasive mistreatment in daily work interactions. This expanded perspective has theoretical implications, calling for an overhaul of current frameworks and measurement tools for workplace mistreatment in public and private sectors. Second, the study highlights the role of the work environment in fostering mistreatment behaviors, resonating with current theories on workplace aggression. The research confirms major contributors such as job stressors and organizational culture, supporting the importance of taking a holistic perspective when researching and addressing mistreatment in the workplace. This study has practical implications for Iranian and international public sector policymakers and managers. The research calls for the immediate review of management practices to eliminate overt and covert mistreatment forms (Mulaphong, 2022). This entails enacting strict codes of conduct and promoting ethical training programs. Additionally, the development of effective reporting mechanisms and intervention strategies is crucial to the development of a safer and more inclusive workplace. The study supports the importance of fostering a positive workplace climate in public sector institutions (Booth et al., 2019). Prioritizing employees’ safety, respect, and empowerment has the potential to reduce mistreatment and its adverse effects. Implementing merit-based policies and having ethical leadership foster a healthier work environment, lowering the levels of employees’ stress as well as interpersonal conflict, in the form of social undermining behavior. Trust and communication have been seen as the factors required for the decrease in mistreatment. Trust is fostered through managers having transparent communication, being transparent in their decision-making processes, as well as being steady in their leadership (Booth et al., 2019).

In addition to our contributions to the mistreatment literature, our model offers novel theoretical insights. Unlike most prior envy research that focuses on the envious actor, our model explicitly centers on how being envied affects the target – effectively shifting attention to the envied employee, a markedly underexplored perspective in this literature (Lee et al., 2018). We introduce perceived injustice as a boundary condition: whereas past studies have emphasized mediators (such as negative emotions or coping strategies), we propose that injustice perceptions moderate the envy–response link; indeed, only a few prior studies have examined justice in envy, and evidence suggests that high procedural justice can buffer the negative consequences of envy (Duffy et al., 2020). Our approach also applies Social Identity Threat and Organizational Justice perspectives in a non-Western public-sector context characterized by limited promotion opportunities and pervasive fairness concerns. In such a context, being envied poses an acute threat to one’s identity and status – consistent with evidence that a lack of fairness creates feelings of “unappreciation and disconnection,” thereby heightening envy (Li et al., 2021). Importantly, we isolate perceived injustice as the sole moderator to preserve parsimony and clearly delineate its unique boundary role, avoiding the confounding influence of additional moderators.

For employees, the biggest message from this research is the significance of noticing subtle mistreatment actions as well as their emotional effects. Employees can protect themselves from the harmful emotions of envy as well as resentment through emotional resilience as well as positive coping. Building strong professional networks with trust as well as respect, mentorship, as well as self-reflection can neutralize the destructive force of social undermining. In addition, self-reflection as well as maintaining the limelight on personal achievement as compared with the tendency toward comparison with other people can neutralize the emotional stimuli of envy. Employees can also empower themselves through the use of the resources of the organization, such as the mechanisms for complaint as well as support mechanisms, as a countermeasure for mistreatment.

For managers, the study emphasizes the importance of creating a good work culture with the well-being of employees as the priority. Managers can promote open communication, maintain transparent decision-making, and hold regular ethical training to provide a work environment where social undermining is less possible. Managers can also recognize and counter envy-driven behavior by promoting teamwork and introducing clear policies for respectful communication. Building and sustaining a robust mechanism for the reporting of mistreatment behavior and the offering of interventions where needed is key to ensuring a healthy work environment.

This study can be applied in the framework of OB and HRM education to inform students on the need of establishing and preserving a moral and encouraging workplace culture. Case studies grounded on the results of this study can be included into OB/HRM courses to underline the complicated character of workplace abuse and its consequences on mental health of employees. HRM courses can include strategies for managing workplace behavior as jealousy and abuse, so giving students useful tools to handle these issues in actual corporate environments. At last, public-sector companies have to create a culture of moral behavior at all levels, so strengthening ethical values and enhancing organizational performance by lowering mistreatment events and their consequences. This study can be used by teachers to talk on the need of trust and communication in avoiding social undermining and the part emotional intelligence plays in handling interpersonal conflicts.

## 6 Limitations and future research

While this study is a significant contributor, there is the necessity of keeping in perspective its limitation. To begin with, the study applied the cross-sectional approach, giving a snapshot perspective of the correlations among the research variables. Future research could enhance causal inference by adopting longitudinal research designs, allowing for examining causal relationships over time. Second, our reliance on self-report measures may introduce common method bias concerns. Researchers interested in workplace mistreatment could broaden their investigations to encompass covert and overt mistreatment behaviors, exploring diverse data sources to mitigate such bias.

We acknowledge that by omitting other empirically supported antecedents and controls—such as role clarity, organizational tenure, hierarchical level, job stress, and personality traits—our model provides a limited examination of perceived injustice as the primary boundary condition. While this decision allowed us to draw a clean connection between envy and social undermining, it necessarily leaves unanswered whether perceived injustice retains its moderating potency when embedded in a fuller nomological network. We therefore encourage future investigations to embed our core model within broader structural equations, testing multiple mediators and control variables concurrently to determine the relative strength and specificity of injustice perceptions in shaping envy-driven mistreatment. Although perceived injustice is seen as a moderator in line with Organizational Justice and Social Identity Threat theories, future studies can look at how envy might indirectly influence changes in justice beliefs. A rigors mediation test looking at the link between envy, perceived unfairness, and social undermining might clarify other processes. Moreover, our study’s generalizability is constrained as data were collected exclusively from a single organization in Iran. Factors associated with this specific context may have influenced our findings. To improve the external validity of future research, it is advisable to include respondents from diverse organizations and cultural backgrounds. Employing alternative data collection techniques like third-party observations can minimize potential common method bias issues.

Data collection occurred between 2021 and 2022, coinciding with the global COVID-19 pandemic, which may have elevated overall mental health levels and confounded our associations. We looked at early and late respondents, who represented various periods of the epidemic, and found no consistent changes in psychological distress or our key variables, suggesting no bias linked to the date of the pandemic. Our main theories also include the relative impacts of workplace envy and injustice on undermining behaviors, which remain important even at higher distress levels. Future research should either replicate the model in a post-pandemic setting or assess and adjust for pandemic-related stressors such health anxiety and lockdown severity to confirm the strength of our results.

## 7 Conclusion

This study investigated the frequently neglected occurrence of social undermining in governmental organizations. Despite often being disregarded, these nuanced kinds of maltreatment profoundly impact employees’ emotional well-being and everyday interactions. Disclosing these clandestine activities has improved our comprehension of how subversion undermines trust, reduces morale, and ultimately threatens organizational efficacy. The findings highlight the imperative for public-sector leaders to broaden their attention beyond explicit abuse and harassment to encompass the more subtle mechanisms that foster resentment and disengagement. Creating a constructive workplace culture, defined by the implementation and perception of fairness, promotes employee well-being and boosts overall performance. This study advocates for a refinement of Organizational Justice Theory. If perceptions of fairness are essential to workers’ understanding and responses to undermining, then models of unproductive work behavior should more comprehensively incorporate the target’s subjective experience. Public administrators at the policy level should consider implementing early detection systems for envy-driven abuse, establishing clear and transparent decision-making processes, and providing cross-cultural fairness training. These actions may mitigate the adverse effects of social undermining and foster environments conducive to creativity and teamwork.

## Data Availability

The raw data supporting the conclusions of this article will be made available by the authors, without undue reservation.
